# Supplementary material for: Hybrid Models and Biological Model Reduction with PyDSTool
Source: PLoS Comput Biol. 2012 Aug 9;8(8):e1002628. doi: 10.1371/journal.pcbi.1002628 (PMC3415397; doi:10.1371/journal.pcbi.1002628)
Supplement: Text S4 — Complete source code for the PyDSTool package (version 0.88.120504). Includes API documentation and help files linking to web pages. This file is identical to the current public release on Sourceforge.net. (ZIP) [file pcbi.1002628.s004.zip › PyDSTool/html/PyDSTool.Toolbox.phaseplane.fixedpoint_2D-class.html]

xml version="1.0" encoding="ascii"?


PyDSTool.Toolbox.phaseplane.fixedpoint\_2D


| Home | Trees | Indices | Help | PyDSTool |
| --- | --- | --- | --- | --- |

Package PyDSTool ::
Package Toolbox ::
Module phaseplane ::
Class fixedpoint\_2D

[hide private]

[frames] | no frames]

# Class fixedpoint\_2D

source code

```
   object --+    
            |    
fixedpoint_nD --+
                |
               fixedpoint_2D
```

---


|  |  |
| --- | --- |
| Instance Methods | [hide private] |

|  |  |
| --- | --- |
| \_\_init\_\_(self, gen, pt, coords=None, jac=None, description=`'``'`, normord=2, eps=1e-12)  pt must have same dimension as generator, but if a sub-system is being analyzed then specify the sub-system's variables using the coords argument. | source code |

|  |  |
| --- | --- |
| \_ensure\_jac(self, jac) | source code |

|  |  |
| --- | --- |
| \_classify(self) | source code |

|  |  |
| --- | --- |
| toarray(self, restrict\_to\_coords=None)  restrict\_to\_coords option of ordered coordinate names (default None) ensures the return array only contains values of those coordinates in that order. | source code |

**Inherited from `fixedpoint_nD`**:
`__getitem__`,
`__setitem__`

**Inherited from `fixedpoint_nD`** (private):
`_get_eigen`

**Inherited from `object`**:
`__delattr__`,
`__getattribute__`,
`__hash__`,
`__new__`,
`__reduce__`,
`__reduce_ex__`,
`__repr__`,
`__setattr__`,
`__str__`


|  |  |
| --- | --- |
| Class Variables | [hide private] |

**Inherited from `fixedpoint_nD`** (private):
`_classifcations`,
`_stability`


|  |  |
| --- | --- |
| Properties | [hide private] |

**Inherited from `object`**:
`__class__`


|  |  |
| --- | --- |
| Method Details | [hide private] |

|  |  |
| --- | --- |
| \_\_init\_\_(self, gen, pt, coords=None, jac=None, description=`'``'`, normord=2, eps=1e-12)  *(Constructor)* | source code |

pt must have same dimension as generator, but if a sub-system is being
analyzed then specify the sub-system's variables using the coords
argument.

Overrides: object.\_\_init\_\_: *(inherited documentation)*

|  |  |
| --- | --- |
| \_ensure\_jac(self, jac) | source code |

Overrides: fixedpoint\_nD.\_ensure\_jac

|  |  |
| --- | --- |
| \_classify(self) | source code |

Overrides: fixedpoint\_nD.\_classify

|  |  |
| --- | --- |
| toarray(self, restrict\_to\_coords=None) | source code |

restrict\_to\_coords option of ordered coordinate names (default None)
ensures the return array only contains values of those coordinates in
that order. Useful for interfacing with plotting commands.

  


| Home | Trees | Indices | Help | PyDSTool |
| --- | --- | --- | --- | --- |

|  |  |
| --- | --- |
| Generated by Epydoc 3.0.1 on Fri May 4 15:24:09 2012 | http://epydoc.sourceforge.net |
